# Antimalarial Potency of *Tinospora crispa* Stem Extract and Artesunate Against *Plasmodium berghei*: A Study on Combination Therapy

**DOI:** 10.1155/jotm/6681227

**Published:** 2026-04-24

**Authors:** Sakaewan Ounjaijean, Voravuth Somsak

**Affiliations:** ^1^ Research Institute for Health Sciences, Chiang Mai University, Chiang Mai, 50200, Thailand, cmu.ac.th; ^2^ School of Allied Health Sciences, Walailak University, Nakhon Si Thammarat, 80160, Thailand, wu.ac.th; ^3^ Research Excellence Center for Innovation and Health Products, Walailak University, Nakhon Si Thammarat, 80160, Thailand, wu.ac.th

## Abstract

Malaria continues to be a significant global health burden, with over 249 million cases and 619,000 deaths reported in 2022, primarily affecting tropical and subtropical regions. The emergence of drug‐resistant strains of *Plasmodium falciparum* has reduced the efficacy of artemisinin‐based combination therapies (ACTs), necessitating the exploration of alternative treatment strategies. This study investigates the antimalarial efficacy of *Tinospora crispa* extract (TCE) and artesunate (ART) against *Plasmodium berghei* ANKA infection in ICR mice. Both ART and TCE demonstrated dose‐dependent antimalarial activity, with effective doses (ED_50_) of 200.31 mg/kg and 1.96 mg/kg, respectively. The combination of ART and TCE exhibited synergistic effects, achieving significantly higher parasitemia inhibition compared to monotherapy, with combination index (CI) values < 1 at ED_50_, ED_50/2_, and ED_50/4_. Furthermore, combination therapy provided enhanced protection against packed cell volume (PCV) reduction and body weight (BW) loss, along with prolonged mean survival time (MST) compared to untreated and single‐treatment groups. These findings highlight the potential of combining TCE with ART as a novel antimalarial strategy, offering synergistic benefits that may mitigate drug resistance and improve treatment outcomes. Further studies are warranted to explore the molecular mechanisms underlying these interactions and to evaluate the efficacy of this combination in clinical settings.

## 1. Introduction

Malaria remains a global public health challenge, particularly in tropical and subtropical regions, causing morbidity and mortality, especially among vulnerable populations, such as children and pregnant women. There were approximately 249 million cases reported in 2022, an increase from prior years. This disease caused over 619,000 deaths worldwide, with more than 70% of these cases occurring in African countries [1]. The causative agents of malaria are *Plasmodium* parasites, with *Plasmodium falciparum* being the most lethal species in humans [2]. The persistent rise in cases reflects challenges posed by emerging drug resistance and environmental changes, which facilitate mosquito breeding and malaria transmission [3]. Despite intensified efforts through prevention campaigns, such as insecticide‐treated bed nets (ITNs), malaria control still requires innovative solutions to achieve elimination targets [4, 5].

Artesunate (ART), a derivative of artemisinin, is widely used as a frontline antimalarial due to its rapid parasite clearance and low toxicity. Artemisinin‐based combination therapies (ACTs) remain the frontline treatment for malaria [6]. However, recent studies highlight growing concerns over artemisinin resistance, especially in Southeast Asia and Africa, which threatens the efficacy of these therapies [7]. As a result, research into alternative or complementary therapies, including combination therapies, has gained momentum to preserve ACT effectiveness and address drug resistance. One promising complementary approach involves the use of natural plant extracts. Natural products offer promising avenues for drug discovery, as they contain bioactive compounds with diverse pharmacological activities [8]. *Tinospora crispa* has been traditionally used in Southeast Asia for managing fever and inflammatory conditions; however, direct ethnomedical documentation specifically supporting its use for malaria remains limited. Consequently, its antimalarial potential has primarily been explored through preclinical experimental studies rather than well‐established traditional practice. [9]. Previous studies have reported that extracts from *T. crispa* exhibit antimalarial, antioxidant, and immunomodulatory properties, making it a potential candidate for combination therapy with existing antimalarial drugs [10]. Extracts of *T. crispa* have shown inhibitory effects on the *P. falciparum* parasite and potential to enhance immune responses, although much of the evidence remains preclinical [11]. Moreover, research on *T. crispa* has demonstrated its promising antimalarial properties, particularly against *Plasmodium berghei* in mouse models. Studies have shown that the ethanol and methanol extracts of *T. crispa* exhibit dose‐dependent antimalarial activity, with higher doses leading to significant inhibition of parasitemia [12–14]. Despite these findings, no studies to date have specifically explored the combination of TCE with ART, a key drug in ACTs. Although several studies have reported antimalarial activity of *T. crispa* extracts (TCEs) in vitro, such models do not capture host–parasite interactions, immune modulation, anemia development, or survival outcomes, which are critical determinants of antimalarial efficacy in vivo. Moreover, the pharmacodynamic behavior of complex plant extracts cannot be reliably extrapolated from in vitro systems alone. Therefore, an in vivo *P. berghei* mouse model was employed in this study to evaluate not only parasitemia suppression but also physiological and survival parameters, providing a more integrated assessment of therapeutic potential.

This study aims to explore the antimalarial potency of TCE when combined with ART against *P. berghei* infection in murine models. The primary goal is to assess whether combination therapy can enhance parasitemia reduction and improve therapeutic outcomes compared to monotherapy. Evaluating such plant–drug combinations is critical in developing more effective treatment regimens that combat emerging drug resistance and improve malaria management strategies. This study aims to fill this research gap, offering a potential alternative that integrates traditional herbal remedies with modern pharmacological approaches.

## 2. Materials and Methods

### 2.1. *T. crispa* Stem Extraction

*T. crispa* stems were collected from mature plants in Chiang Mai, Thailand. The plant was identified by a botanist, and a voucher specimen, containing both leaves and stems, was prepared, pressed, and deposited in the Research Institute for Health Sciences, Chiang Mai University, with the assigned voucher number (WU‐TCS‐552024) for future reference. Healthy, mature stems were selected, washed to remove debris, and cut into small sections before drying in a hot‐air oven at 40°C–50°C for 2–3 days. The dried stems were ground into a fine powder, stored in airtight containers, and extracted using 95% ethanol at a 1:10 ratio (e.g., 100 g of powder with 1000 mL of ethanol). The mixture was incubated on a rotary shaker at 150 rpm for 48–72 h at room temperature, protected from light, and then filtered. The filtrate was concentrated using a rotary evaporator under reduced pressure at 40°C–50°C to remove ethanol, with further drying under a fume hood if necessary. The final extract was stored in amber bottles at 4°C until use [12].

### 2.2. ART Preparation

ART was purchased from Sigma‐Aldrich (St. Louis, MO, USA) to ensure quality and compliance with research‐grade standards. Upon receipt, the ART was stored according to the manufacturer’s instructions at room temperature in a dry, dark environment to maintain stability. For in vivo administration, ART was freshly prepared on each experimental day by dissolving the required amount in 20% Tween‐80 to achieve the desired concentration. The solution was vortexed to ensure complete dissolution and administered to the experimental mice via oral gavage [15].

### 2.3. ICR Mice

ICR mice, a widely used outbred strain, were obtained from Nomura Siam International Co., Ltd. (Bangkok, Thailand). All animals were male, 4–6 weeks old, and weighed 20–25 g at the start of the experiment. The mice were housed in groups of 3–5 per cage under standard laboratory conditions, including a temperature of 22°C–25°C, 12:12‐h light–dark cycle, and 40%–60% relative humidity. They were provided with standard rodent chows and water *ad libitum*. All animals were acclimatized to the laboratory environment for at least 7 days before the start of the experiments. Health status was monitored daily throughout the study to ensure animal welfare. Euthanasia of experimental animals was carried out in accordance with institutional animal care and guidelines. An overdose of Zoletil 50 (tiletamine–zolazepam, 100 mg/kg, intraperitoneal injection) was administered to ensure rapid and humane termination. Death was confirmed by the permanent cessation of heartbeat and the absence of corneal and pedal reflexes, in compliance with accepted criteria for humane euthanasia. Handling, housing, and all experimental procedures followed the guidelines set by the Walailak University Animal Care and Use Committee (WU‐ACUC), with ethical approval number WU‐ACUC‐67063.

### 2.4. *P. berghei*

The malaria parasite *Plasmodium berghei* ANKA (PbANKA), a rodent‐specific strain commonly used to model human malaria in experimental studies, was obtained from the Malaria Research and Reference Reagent Resource Center (MR4, BEI Resources, Manassas, VA, USA). The parasite was maintained through serial passage in ICR mice to ensure viability and infectivity. Infected blood samples were collected from donor mice at the peak of parasitemia (typically 20%–30%) by cardiac puncture under anesthesia with 2% isoflurane and diluted with phosphate‐buffered saline (PBS) to achieve the desired concentration of parasitized red blood cells. For experimental infections, mice were inoculated intraperitoneally with 1 × 10^7^ infected red blood cells (iRBC) suspended in 200 μL of PBS. Parasitemia was monitored regularly by preparing thin blood smears, staining them with Giemsa, and examining them under a light microscope to confirm successful infection and progression of the parasite [16].

(1) Parasitemia %=Number of iRBC×100Total number of RBC counted.

### 2.5. Effective Dose Determination

The effective doses (ED_50_) of antimalarial agents were determined using the 4‐day suppressive test model in mice infected with PbANKA [17]. Mice were divided into treatment groups, with each group receiving either ART at doses of 0.1, 1.0, 5.0, 10, and 20 mg/kg or TCE at doses of 50, 100, 200, 400, and 800 mg/kg. Treatments were administered via oral gavage once daily for four consecutive days, starting 24 h after infection. Parasitemia levels were assessed on day 4 postinfection by preparing thin blood smears from tail blood, staining them with Giemsa, and counting the number of iRBC under a light microscope. The percentage of inhibition of parasitemia was calculated using the formula as follows:

(2) inhibition %=mean parasitemia of control−mean parasitemia of treatment×100mean parasitemia of control.

This calculation enabled the determination of the effectiveness of each treatment dose in reducing parasitemia compared to the control group. The ED_50_ were identified based on the dose–response relationship and the corresponding percent inhibition values, allowing for the evaluation of the potency of ART and TCE in suppressing *P. berghei* infection in the experimental model.

### 2.6. Combination Antimalarial Evaluation

The investigation of combination antimalarial therapy was conducted using the 4‐day suppressive test model in mice infected with PbANKA [17]. Mice were divided into various treatment groups to evaluate the efficacy of the combination of ART and TCE. The ED_50_ for each compound were determined from preliminary dose–response studies, with the selected doses for combination therapy being ED_50_, ED_50/2_, ED_50/4_, and ED_50/8_. For the combination therapy, groups were treated with TCE and ART in a fixed ratio of 1:1 based on their respective ED_50_ [18]. This fixed‐ratio design was selected as it represents a standard approach in pharmacodynamic interaction studies, allowing for balanced contribution of each agent and facilitating the evaluation of synergistic, additive, or antagonistic effects through combination index (CI) analysis. In addition, this approach enables systematic dose reduction while maintaining proportional exposure to both compounds. Mice received the combination treatment once daily for four consecutive days, starting 24 h postinfection. Parasitemia was assessed on day 4 by preparing thin blood smears, staining them with Giemsa, and calculating the percentage of iRBC. The inhibition of parasitemia was subsequently computed. Additionally, packed cell volume (PCV) and body weight (BW) were monitored to assess the impact of treatments on anemia and general health. Changes in PCV were measured using microhematocrit tubes, while BW was recorded using a digital balance. Moreover, mean survival time (MST) was calculated for each treatment group to evaluate the overall effectiveness of the combination therapy in prolonging survival.

### 2.7. PCV Measurement

The PCV was measured to assess the degree of anemia in experimental mice infected with PbANKA. Blood samples were collected from the tail vein of each mouse into heparinized microcapillary tubes. The tubes were sealed with clay at one end and centrifuged at 12,000 rpm for 5 min using a microhematocrit centrifuge. After centrifugation, the proportion of RBC (PCV) was measured as a percentage of the total blood volume using a hematocrit reader. PCV values were recorded at baseline (pre‐infection) and monitored at specific time points postinfection to evaluate the effectiveness of the treatments in preventing or mitigating malaria‐induced anemia. A decline in PCV indicated the progression of infection, while stabilization or recovery suggested a positive therapeutic effect.

### 2.8. BW Measurement

The BW of each mouse was measured regularly to monitor the health status and potential toxic effects of the treatments throughout the study. Weights were recorded using a digital balance with a precision of 0.01 g. Baseline BW was measured before infection with PbANKA, and subsequent measurements were taken daily or at specific time points postinfection and treatment. Changes in BW were used as an indicator of disease progression, therapeutic efficacy, and potential side effects of the treatments. Weight loss was interpreted as a sign of illness or toxicity, while stable or increased BW indicated improved health or recovery.

### 2.9. MST Determination

MST was assessed to evaluate the efficacy of treatments in prolonging the lifespan of mice infected with PbANKA. Mice were observed daily throughout the experimental period (30 days), and the number of days from parasite inoculation until death was recorded for each animal. A longer MST indicated greater treatment efficacy in delaying death and improving survival. The MST for each group was calculated using the formula as follows:

(3) MST=∑survival time of each mouse daysnumber of mice in the group.

### 2.10. Statistics

All data were analyzed using GraphPad Prism Version 10.1.1 (GraphPad Software Inc., San Diego, California, USA). Results were expressed as mean ± standard error of the mean (SEM) for each experimental group. One‐way analysis of variance (ANOVA) was performed to compare differences among multiple groups, followed by Tukey’s post hoc test to identify specific group differences. A *p*‐value of < 0.05 was considered statistically significant. Nonlinear regression analysis with a variable slope model was used to evaluate dose–response relationships and determine ED_50_ values of the treatments. Moreover, combination therapy interactions between ART and TCE were analyzed using CompuSyn software (ComboSyn, Inc., USA) to determine whether the combination exhibited synergistic, additive, or antagonistic effects. The CI was calculated, with CI < 1 indicating synergy, CI = 1 indicating an additive effect, and CI > 1 indicating antagonism.

## 3. Results

### 3.1. Propagation of *P. berghei* ANKA Infection in Mice

Figure 1 demonstrates the propagation of PbANKA infection in ICR mice, showcasing the typical progression of malaria in this model. Parasitemia levels increased steadily from day 2 (1.67 ± 0.55%) to day 14 postinfection (58.59 ± 6.28%), confirming the successful establishment and proliferation of the parasite within RBC. Concurrently with the rise in parasitemia, markedly declines in PCV and BW were observed, indicating the progressive disease burden induced by PbANKA infection. These observations align with previous findings from experimental malaria studies (Figure 1(a)).

FIGURE 1Propagation of *Plasmodium berghei* ANKA infection in mice. ICR mice were intraperitoneally inoculated with 1 × 10^7^ infected red blood cells. (a) Parasitemia, packed cell volume (PCV), and body weight (BW) were monitored daily throughout the experimental period. (b–c) The correlations between parasitemia and PCV, as well as parasitemia and BW, were analyzed. (d) Cumulative survival rates of infected mice were recorded. Data are presented as the mean ± standard error of the mean (SEM), with five mice per group.

**Figure:**
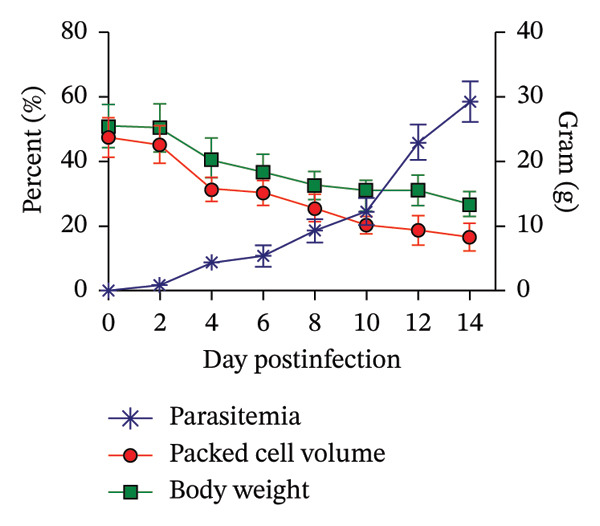
(a)

**Figure:**
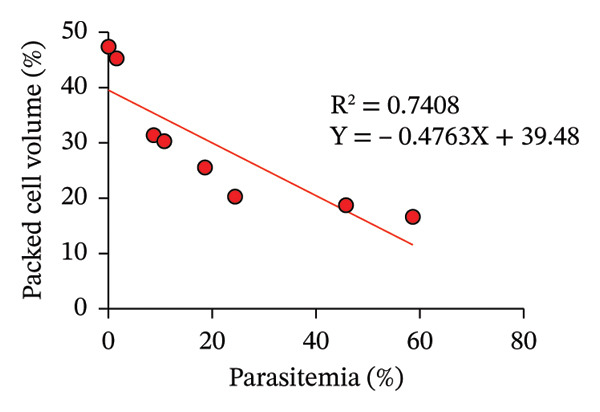
(b)

**Figure:**
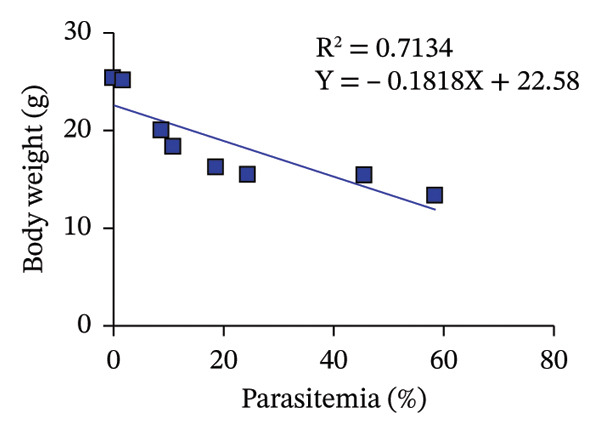
(c)

**Figure:**
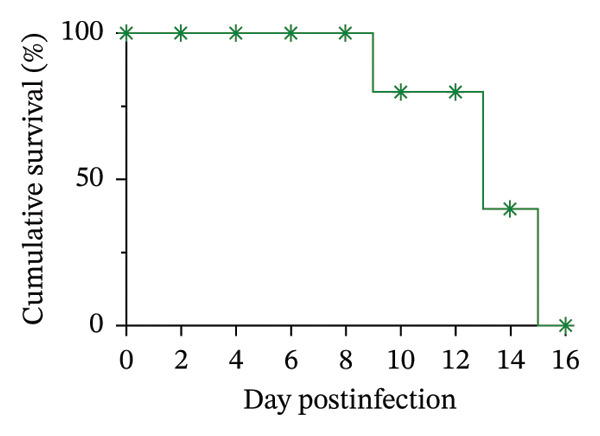
(d)

The correlations between parasitemia and physiological parameters are depicted in Figures 1(b) and 1(c). A strong inverse relationship was identified between parasitemia and PCV (*R* ^2^ = 0.7408), suggesting that higher parasitemia levels are associated with severe anemia, a hallmark of malaria pathogenesis. Similarly, parasitemia exhibited a negative correlation with BW (*R* ^2^ = 0.7134), indicating that increasing parasite loads contribute to substantial weight loss. These trends underscore the impact of PbANKA infection on both hematological and metabolic health, reinforcing the value of monitoring PCV and BW alongside parasitemia as reliable indicators of disease progression and therapeutic efficacy.

Figure 1(d) presents the cumulative survival curve of infected mice, with a calculated MST of 13.6 ± 2.07 days. This outcome highlights the rapid progression of PbANKA infection, as untreated mice typically succumb to the disease within two weeks. The limited survival reflects the combined effects of rising parasitemia, progressive anemia (evidenced by declining PCV), and BW loss, which collectively contribute to severe morbidity and eventual mortality. These results emphasize the importance of early intervention and provide a benchmark for assessing the efficacy of antimalarial treatments in preclinical models.

### 3.2. Effective Doses of ART and TCE Against *P. berghei* ANKA Infection in Mice

Figure 2 illustrates the antimalarial activities of ART and TCE against PbANKA infection in ICR mice. ART exhibited significant (*p* < 0.05) antimalarial efficacy in a dose‐dependent manner at 1, 5, 10, and 20 mg/kg, achieving parasitemia inhibition rates of 40%, 59.52%, 95%, and 100%, respectively (Figure 2(a)). Notably, TCE also demonstrated significant (*p* < 0.05) antimalarial activity, with inhibition increasing in a dose‐dependent fashion. At doses of 200, 400, and 800 mg/kg, TCE achieved 52.02%, 80%, and 82.02% inhibition of parasitemia, respectively. The ED_50_ values, representing the dose required to achieve 50% parasitemia inhibition, were calculated for both treatments (Figure 2(b)). ART exhibited an ED_50_ of 1.96 mg/kg (∼2 mg/kg), while TCE showed an ED_50_ of 200.31 mg/kg (∼200 mg/kg). These results highlight the potent antimalarial properties of ART at low doses and the potential of TCE as an alternative or adjunct therapy, particularly given its dose‐dependent inhibitory effects. Further exploration of their combination could provide new insights into developing synergistic treatment strategies.

FIGURE 2Determination of the ED_50_ values of artesunate (ART) and *Tinospora crispa* extract (TCE) against *Plasmodium berghei* ANKA infection in ICR mice. Mice were intraperitoneally inoculated with 1 × 10^7^ infected red blood cells and subsequently treated orally with TCE at doses ranging from 50 to 800 mg/kg for four consecutive days. ART, at doses of 0.1–20 mg/kg, was used as a positive control. Parasitemia and percent inhibition were then estimated. Data are expressed as the mean ± standard error of the mean (SEM), with five mice per group. Statistical significance was denoted as ^∗^ *p* < 0.05, ^∗∗^ *p* < 0.01, and ^∗∗∗^ *p* < 0.001, compared to the untreated (UN) group.

**Figure:**
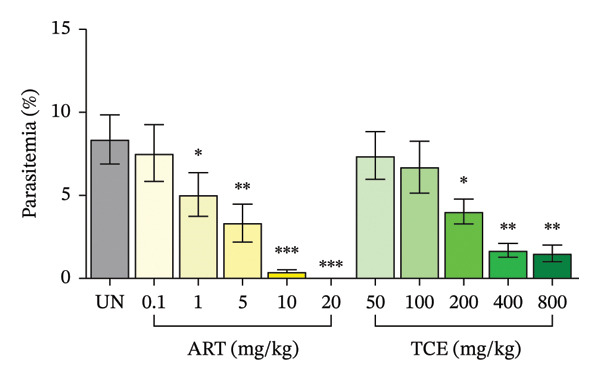
(a)

**Figure:**
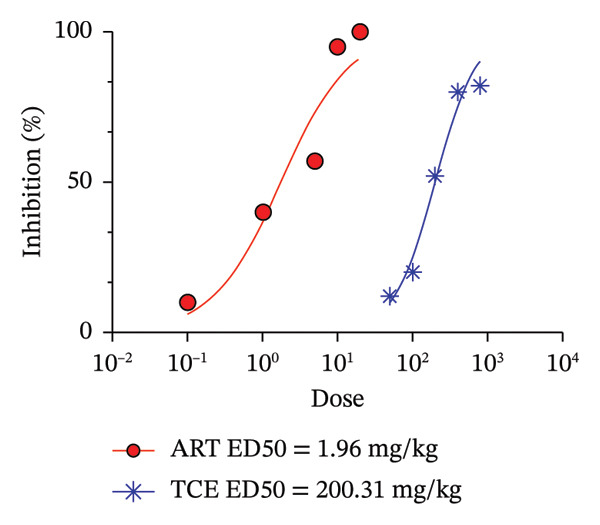
(b)

### 3.3. Combination Treatment of ART and TCE Against *P. berghei* ANKA Infection in Mice

Figure 3(a) demonstrates the antimalarial efficacy of the combination therapy involving ART and TCE at various ED_50_ ratios. The combinations at ED_50_, ED_50/2_, and ED_50/4_ exhibited significant inhibition of parasitemia, achieving 90.01%, 93.15%, and 85.04% inhibition, respectively. These results were statistically significant compared to the untreated control group (*p* < 0.001) and the groups receiving single‐agent ART or TCE treatments (*p* < 0.05).

FIGURE 3Evaluation of combination therapy with artesunate (ART) and *Tinospora crispa* extract (TCE) against *Plasmodium berghei* ANKA infection in mice. ICR mice were intraperitoneally inoculated with 1 × 10^7^ infected red blood cells. Combination treatments of ART and TCE at a 1:1 ratio were administered orally for four consecutive days at doses equivalent to ED_50_, ED_50/2_, ED_50/4_, and ED_50/8_. (a–b) Parasitemia and percentage inhibition were assessed. (c–d) Packed cell volume (PCV) and body weight were also monitored. Data are presented as the mean ± standard error of the mean (SEM), with five mice per group. Statistical significance was indicated as ^∗^ *p* < 0.05, ^∗∗^ *p* < 0.01, and ^∗∗∗^ *p* < 0.001, compared to either the untreated (UN) or healthy (H) group.

**Figure:**
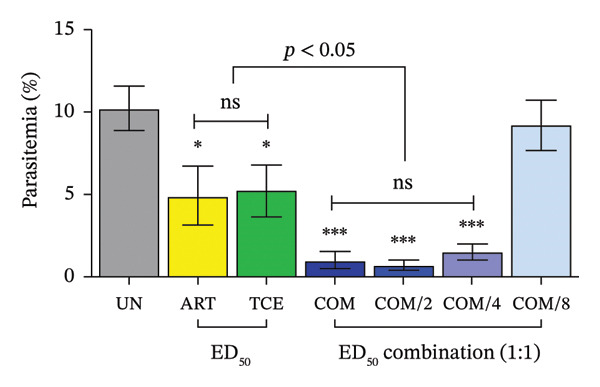
(a)

**Figure:**
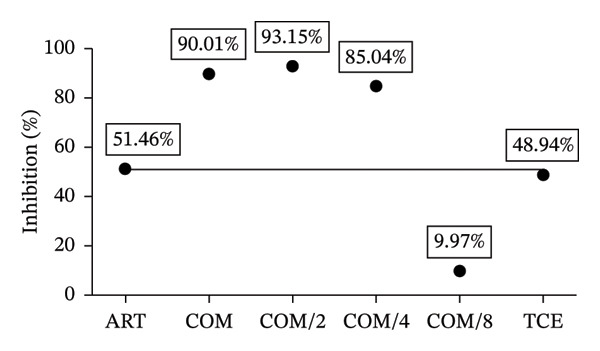
(b)

**Figure:**
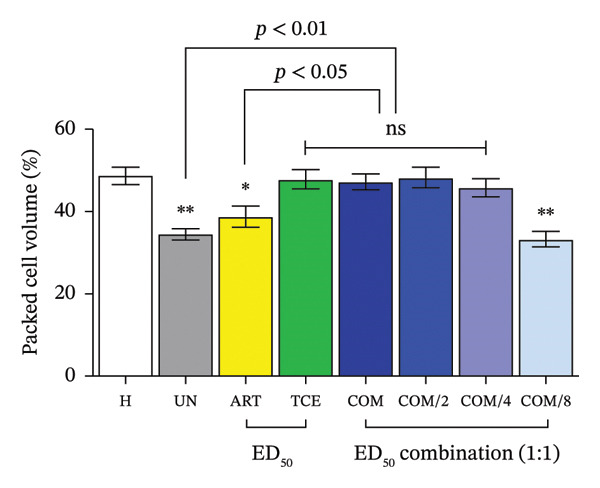
(c)

**Figure:**
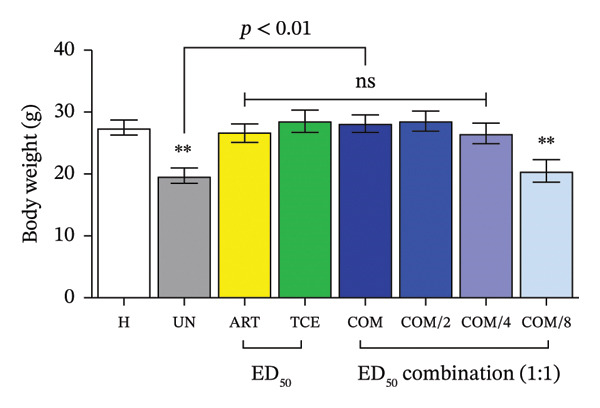
(d)

The CI analysis, shown in Figure 3(b) and Table 1, revealed synergistic interactions between ART and TCE at ED_50_, ED_50/2_, and ED_50/4_ with CI values of 0.55405, 0.15561, and 0.19861, respectively. These CI values, all less than 1, indicate a synergistic effect, suggesting that the combination therapy is more effective than the individual agents alone. However, at the lowest ratio (ED_50/8_), the observed CI value suggested an antagonistic interaction, indicating a potential reduction in efficacy at this lower combination dose.

**TABLE 1:** Combination index of ART combined with TCE against PbANKA infection.

Antimalarial test		Dose (mg/kg)		CI value
		ART	TCE	
Combination (1:1)	COM (ED_50_)	2.00	200	0.55405^a^
	COM/2 (ED_50/2_)	1.00	100	0.15561^b^
	COM/4 (ED_50/4_)	0.50	50	0.19861^b^
	COM/8 (ED_50/8_)	0.25	25	2.34109^b^

^a^CI < 1, synergism.

^b^CI > 1, antagonism.

### 3.4. Effects of Combination Treatment of ART and TCE on PCV, BW, and MST in *P. berghei* ANKA Infection in Mice

A significant reduction in PCV and BW was observed in PbANKA‐infected mice (*p* < 0.01), highlighting the physiological impact of the infection (Figures 3(c) and 3(d)). Mice treated with ART alone exhibited a marked decrease in PCV, which was statistically significant (*p* < 0.05). As anticipated, combination therapy with ART and TCE at ED_50_, ED_50/2_, and ED_50/4_ provided significant protection against PCV reduction and BW loss compared to both the untreated group and the group treated with ART alone (*p* < 0.05). These findings suggest that combination therapy offers improved physiological outcomes over monotherapy.

**FIGURE 4:**
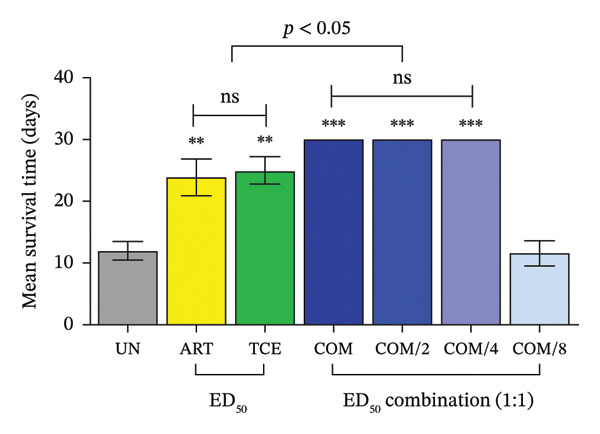
Mean survival time (MST) of *Plasmodium berghei* ANKA‐infected mice following combination treatment with artesunate (ART) and *Tinospora crispa* extract (TCE). ICR mice were intraperitoneally inoculated with 1 × 10^7^ infected red blood cells. Combination treatments of ART and TCE at a 1:1 ratio were administered orally for four consecutive days at doses equivalent to ED_50_, ED_50/2_, ED_50/4_, and ED_50/8_. MST was recorded over a 30‐day period. Data are expressed as the mean ± standard error of the mean (SEM), with five mice per group. Statistical significance was denoted as ^∗∗^ *p* < 0.01 and ^∗∗∗^ *p* < 0.001, compared to the untreated (UN) group.

Additionally, mice receiving combination treatment at ED_50_, ED_50/2_, and ED_50/4_ demonstrated significantly longer MST compared to the untreated group (*p* < 0.001) and those treated with ART or TCE alone (*p* < 0.05), as shown in Figure 4. This extended MST highlights the enhanced therapeutic potential of the ART and TCE combination, reinforcing the value of synergistic dosing strategies in mitigating disease progression and improving survival outcomes in experimental malaria models.

## 4. Discussion

This study investigates the antimalarial efficacy of TCE in combination with ART against PbANKA infection in mouse models. The primary objective is to determine whether combination therapy can achieve superior parasitemia reduction and improved therapeutic outcomes compared to monotherapy with either agent alone. The results revealed significant correlations between parasitemia and physiological parameters, PCV and BW as well as their impact on MST. The inverse relationships observed between parasitemia and PCV, and between parasitemia and BW, reflect the systemic burden of PbANKA infection in mice, underscoring how parasite proliferation drives hematological and metabolic deterioration [19]. The strong negative correlation between parasitemia and PCV (*R* ^2^ = 0.7408) indicates that higher parasite loads correspond to more severe anemia. This aligns with the typical pathology of malaria, where the destruction of infected and noninfected red blood cells, alongside suppression of erythropoiesis, contributes to a decline in hematocrit levels [20]. Anemia is not only a marker of disease severity but also a predictor of mortality in malaria infections, as insufficient oxygen transport impairs organ function and resilience during infection [21, 22]. Similarly, the inverse relationship between parasitemia and BW (*R* ^2^ = 0.7134) suggests that increasing parasite burden leads to significant weight loss. This reduction in BW is likely due to systemic inflammation, reduced food intake, metabolic stress, and impaired energy regulation, which are common in malaria‐infected animals [23, 24]. Weight loss has also been associated with poor prognosis, as it reflects the host’s inability to cope with the physiological demands of the infection [24]. These declines in PCV and BW are further linked to reduced MST. In this study, the MST of untreated PbANKA‐infected mice was 13.6 ± 2.07 days, reflecting the rapid progression of the disease. The combined impact of severe anemia and weight loss accelerates mortality, as both conditions exacerbate organ failure and weaken the host’s ability to mount an immune response. The findings emphasize the importance of monitoring parasitemia, PCV, and BW as key indicators of disease progression and treatment efficacy in malaria studies. These parameters provide a comprehensive picture of the physiological burden imposed by the infection and help evaluate the success of therapeutic interventions. Treatments that can reduce parasitemia, stabilize PCV, and maintain BW are more likely to prolong survival and improve overall health outcomes, as evidenced by the improved MST in groups receiving combination therapy.

Moreover, this study highlights the dose‐dependent antimalarial efficacy of ART and TCE against PbANKA infection, with ED_50_ values of 1.96 mg/kg for ART and 200.31 mg/kg for TCE. ART, known for its rapid action, demonstrated potent parasitemia inhibition at low doses, while TCE also exhibited significant antimalarial activity, albeit at higher doses, suggesting complementary roles in combination therapy. The mechanism of ART involves the generation of reactive oxygen species (ROS) and heme‐iron adducts, leading to oxidative damage in the parasite’s proteins and membranes [6]. ART targets parasite‐infected red blood cells during the asexual blood stage, disrupting essential metabolic processes and causing cell death [25]. Due to this rapid parasite clearance, ART is effective at reducing parasitemia early in infection [26]. However, the emergence of artemisinin resistance has raised concerns, making combination strategies essential for maintaining treatment efficacy. *T. crispa* contains multiple bioactive compounds, including borapetoside, flavonoids, alkaloids, and terpenoids. TCE contains bioactive compounds reported to possess immunomodulatory and antioxidant properties; however, whether these effects directly contribute to the enhanced antimalarial activity observed here requires further mechanistic investigation [9, 10]. Borapetoside has been reported to modulate the immune system by enhancing macrophage activation and cytokine production, contributing to the host’s ability to fight parasitic infections [9, 27–29]. Additionally, TCE’s antioxidant properties may help reduce oxidative stress in infected tissues, mitigating some of the adverse effects caused by malaria [10, 30].

The combination of ART and TCE at ED_50_, ED_50/2_, and ED_50/4_ demonstrated potent antimalarial activity, with significantly higher inhibition of parasitemia compared to individual treatments. These findings indicate a synergistic interaction, where the combination of ART and TCE enhances therapeutic efficacy beyond what each agent achieves independently. The observed synergistic effects may reflect complementary pharmacological actions of ART and TCE; however, the precise molecular mechanisms underlying this interaction were not directly investigated in this study and therefore remain speculative [6, 10]. Interestingly, the combination at ED_50/8_ did not exhibit the same antimalarial potency, suggesting that insufficient doses of both ART and TCE may compromise therapeutic effectiveness. At this suboptimal dose, the ART concentration may fall below the threshold required for rapid parasite clearance, and the immunomodulatory effects of TCE may not be strong enough to sustain parasite suppression. This underscores the importance of maintaining effective dosing levels to achieve synergy; otherwise, the reduced doses may result in antagonistic interactions, as reflected by the observed CI value > 1. The dose‐dependent nature of this combination therapy demonstrates the critical balance needed for achieving synergy. While ED_50_, ED_50/2_, and ED_50/4_ showed robust inhibition, the failure of ED_50/8_ highlights the risk of underdosing, which may not only diminish efficacy but also encourage the emergence of resistant parasites. These findings emphasize that optimal dosing strategies are crucial to maximize the benefits of combination therapies.

The combination of ART and TCE not only demonstrated superior antimalarial activity by reducing parasitemia but also significantly improved key physiological parameters, including PCV, BW, and MST. The combination of ART and TCE at effective doses (ED_50_, ED_50/2_, and ED_50/4_) provided significant protection against the decline in PCV compared to monotherapy or untreated groups. ART’s rapid reduction of parasitemia likely minimized red blood cell destruction, while TCE’s antioxidant and immune‐modulating properties, linked to compounds, such as borapetoside, may have supported erythropoiesis and mitigated oxidative damage to red cells [10, 31]. This suggests that the combination therapy not only suppresses parasite growth but also helps restore hematological health, reducing the severity of malaria‐induced anemia. Malaria infections often lead to weight loss due to systemic inflammation, reduced appetite, and metabolic stress. In this study, the combination therapy at higher doses (ED_50_, ED_50/2_, and ED_50/4_) effectively mitigated BW loss, reflecting an improvement in the general health and metabolic status of the treated mice. ART’s ability to reduce parasitemia likely alleviated systemic inflammation, while TCE may have contributed by improving immune function and reducing oxidative stress, promoting recovery and weight maintenance [16, 32, 33]. In contrast, the lowest combination dose (ED_50/8_) did not provide the same level of protection, suggesting that suboptimal dosing does not sustain the necessary therapeutic effect. Moreover, the combination therapy significantly extended the MST of infected mice compared to monotherapy and untreated controls, with the highest doses achieving the most substantial survival benefits. The prolonged MST indicates that the synergy between ART and TCE not only suppresses parasitemia more effectively but also enhances the host’s resilience to the infection. Enhanced survival is likely the result of multiple factors, including improved PCV, stabilized BW, and reduced oxidative and inflammatory damage, highlighting the holistic benefits of the combination approach.

Overall, this study contributes to the growing body of evidence supporting the integration of plant‐based therapies with conventional antimalarial drugs. The combination of ART and TCE offers a promising approach to malaria treatment by enhancing parasitemia suppression and improving clinical outcomes. Several limitations should be acknowledged. This study did not directly examine molecular targets or immune pathways involved in the observed synergistic effects. Furthermore, although the *P. berghei* mouse model provides valuable translational insight, it does not fully replicate human malaria. Therefore, these findings should be interpreted as preclinical evidence, and further mechanistic, pharmacokinetic, and clinical studies are required before therapeutic application can be considered.

## 5. Conclusion

This study confirms the antimalarial potential of TCE in combination with ART against PbANKA infection. Both treatments showed dose‐dependent inhibition of parasitemia, with the combination therapy demonstrating synergistic effects at multiple dose levels. The combination significantly improved physiological outcomes, including preservation of PCV, mitigation of BW loss, and extension of MST compared to monotherapy or untreated controls. These findings suggest that the ART and TCE combination represents a promising preclinical strategy for enhancing antimalarial efficacy. However, additional mechanistic and translational studies are required to determine its relevance to ART resistance and potential clinical application. Future studies should focus on identifying the specific bioactive components in TCE responsible for its antimalarial activity and elucidating their molecular interactions with ART. Additionally, pharmacokinetic studies are essential to optimize dosing strategies and assess the long‐term safety of this combination. This research offers a promising avenue for developing novel therapeutic approaches that integrate traditional herbal remedies with modern pharmacotherapy to address the challenges of drug‐resistant malaria.

## Author Contributions

Sakaewan Ounjaijean was responsible for experimental design, laboratory work, data analysis, manuscript drafting, revisions, and finalization. Voravuth Somsak supervised and conceptualized the research, secured funding, provided overall project oversight, managed correspondence, contributed to manuscript preparation, and granted final approval.

## Funding

This work was supported by the Research Institute for Health Sciences, Chiang Mai University, Chiang Mai, Thailand.

## Disclosure

Both authors have reviewed and approved the final version of the manuscript for publication.

## Ethics Statement

All animal procedures in this study were conducted in strict accordance with the guidelines and regulations for the care and use of laboratory animals as outlined by the Walailak University Animal Care and Use Committee (WU‐ACUC). Ethical approval (WU‐ACUC‐67063) for the study was obtained from the WU‐ACUC before the commencement of the experiments. Every effort was made to minimize animal suffering, reduce the number of animals used, and ensure that humane endpoints were implemented whenever necessary. All handling and experimental procedures were performed by trained personnel to ensure the welfare and ethical treatment of the animals. Moreover, this study also adhered to the Animal Research: Reporting of In Vivo Experiments (ARRIVE) guidelines to ensure transparent and comprehensive reporting of animal research. Detailed experimental protocols, including animal care, handling, and experimental design, were structured to align with these guidelines. The ARRIVE checklist was utilized during manuscript preparation, and the relevant information is provided to enable critical assessment of the study’s reliability [34].

## Conflicts of Interest

The authors declare no conflicts of interest.

## Data Availability

The data that support the findings of this study are openly available in Figshare at https://figshare.com/s/e3d0f17ebdb148a05ba4, reference number 10.6084/m9.figshare.27328308.
